# The Moderated Mediating Effect of Hope, Self-Efficacy and Resilience in the Relationship between Post-Traumatic Growth and Mental Health during the COVID-19 Pandemic

**DOI:** 10.3390/healthcare10061091

**Published:** 2022-06-12

**Authors:** Donatella Di Corrado, Benedetta Muzii, Paola Magnano, Marinella Coco, Rosamaria La Paglia, Nelson Mauro Maldonato

**Affiliations:** 1 Faculty of Human and Social Sciences, Kore University of Enna, Cittadella Universitaria, 94100 Enna, Italy; paola.magnano@unikore.it (P.M.); rosamarialapaglia@gmail.com (R.L.P.); 2 Department of Humanistic Studies, University of Naples Federico II, 80138 Naples, Italy; benedetta.muzii@unina.it; 3 Department of Biomedical and Biotechnological Sciences, University of Catania, 95123 Catania, Italy; marinella.coco@gmail.com; 4 Department of Neuroscience and Reproductive and Odontostomatological Sciences, University of Naples Federico II, 80138 Naples, Italy; nelsonmauro.maldonato@unina.it

**Keywords:** post-traumatic growth, well-being, hope, mental health, COVID-19

## Abstract

As a major life trauma, COVID-19 had negative impacts on psychological well-being. The aim of this study was to test and verify the mediation of resilience, hope and self-efficacy and to analyze the moderating effect of gender and COVID-19 contagion on the association between symptoms of anxiety, stress and depression, and post-traumatic growth among adults during COVID-19. A cross-sectional study was conducted via an online survey with self-administered questionnaires. The Depression, Anxiety, and Stress Scale, the Post-traumatic Growth Inventory, the Resilience Scale, the General Self-Efficacy Scale, and the Comprehensive State Hope Scale were used. A total of 493 *(n* = 262 female and *n =* 231 male) participated in the survey (*M_age_* = 33.40 years, *SD* = 13.41, range = 20 to 60). A multiple mediation model was used to verify the mediating effect of resilience, hope and self-efficacy on the relationship between symptoms of anxiety, stress and depression, and PTG. A moderated mediation model was examined to find and test the moderated effects of gender and COVID-19 contagion on the mediation model. Results showed the indirect effects of anxiety (β = 0.340 (0.120); 95% CI [from 0.128 to 0.587]) and depression (β = 0.222 (0.095); 95% CI [0.048, 0.429]) on PTG trough resilience and hope. Symptoms of anxiety, stress and depression, and self-efficacy were significantly highest in females. Moreover, males and participants with COVID-19 contagion had significantly higher levels of resilience and post-traumatic growth. These findings suggest that hope and resilience, as protective factors, could be an important key to developing an intervention strategy to enhance and improve psychological health during a crisis.

## 1. Introduction

In late December 2019, a life-threatening kind of pneumonia named COVID-19 was attested in Wuhan, China. The number of testified cases quickly increased, declaring this virus a pandemic by World Health Organization. The transmission of COVID-19 occurs predominantly through droplets and strict contact. In order to restrain the spread of the virus, the direct measure of defense was the governments’ protective restrictions. The next steps included full lockdowns of nations, the deletion of travel, restrictions on social congregations, and the closing of educational institutions.

As a major life trauma, COVID-19 had negative impacts on individuals, such as symptoms post-traumatic stress symptoms, anxiety, depression and fear [1,2,3]. The prolonged quarantine in Italy has profoundly transformed the habits of individuals, requiring them to practice “social and physical distancing”. Home confinement, while being a safety measure to avoid human-to-human transmission of the virus, had unintended negative consequences that damagingly impacted individual well-being. According to Rossi et collaborators [4], the general population reported symptoms of anxiety, depression, stress, increased loneliness and sleep disorders. Delmastro et colleagues [5] assessed 18,147 Italian individuals and found high levels of post-traumatic stress symptoms (37.14%), depression (17.3%), anxiety (20.8%) and stress (21.9%), with women expressing a higher impact on their mental health.

Depression is a disorder that diminishes the proper functioning in individuals; it is associated with loss of interest, loss of life enjoyment and energy [6]. Anxiety is the natural reaction to danger; anxiety disorder is manifested when this reaction is related to a higher level of arousal and inefficient coping strategies [7]. Stress is the feeling of being overwhelmed or unable to cope with mental or emotional pressure [8]. Despite numerous studies that have examined the negative psychological impact of COVID-19, maintaining positive functioning may be possible as a normative response in the presence of emotional distress [9,10].

Furthermore, many individuals experience positive changes, or post-traumatic growth (PTG), referring to an optimal adaptation process that people consider as a result of their cognitive efforts to deal with their disastrous events. Through the cognitive and emotional fights following the trauma, individuals begin to rebuild their own growth, which can lead to well-being and adjustment [11]. Therefore, it is the cognitive process used to adapt after the trauma that promotes growth in numerous areas of life (interpersonal relationships, new possibilities, personal power, mental changes, life enjoyment) [12]. Post-traumatic growth refers to some natural resources that generate coping skills and promote well-being and adaptability against stressors, including self-efficacy, hope and resilience [13].

Hopeful individuals are considered to experience positive emotions and tend to view difficulties in success as challenges that need to be overcome rather than stress factors. People high in positive mental well-being have self-efficacy to achieve their goals and the capability to influence others. They also show resilience because they have the necessary skills and resources to regain quickly from stressful situations [14].

Resilience, self-efficacy and hope have been studied either separately, as part of the same conceptual framework, or as protective moderating or mediating constructs in relation to symptoms of anxiety, stress and depression.

### 1.1. Self-Efficacy

Self-efficacy is a key variable in the social cognitive theory of Bandura [15], which is defined as individuals’ belief in their ability to establish motivational, cognitive and behavioral resources to complete situational demands. The theory showed that perceived self-efficacy affects coping efforts and the stronger the self-efficacy, the more active the efforts, indicating self-efficacy as one of the most important components of success and compromising. In this context, self-efficacy beliefs directly or indirectly affect the behaviors of people, their level of endurance against worries and failures and the stress they face while dealing with environmental difficulties [16].

Different studies confirmed its important role in adaptive confrontation styles in stressful situations, highlighting people who have high levels of self-efficacy have control over their own thoughts and show more stability, offering psychological resilience in achieving goals [17,18].

The literature has investigated this topic widely. To summarize, self-efficacy was found to be positively related to subjective well-being [19], life satisfaction [20] and hope [21]. On the other hand, self-efficacy was negatively associated with anxiety [22], fear [23] and depressive symptoms [24].

### 1.2. Hope

Hope has a special role in human life. Hope is generally defined as the extent to which people possess a positive faith for their future for themselves, being able to cope better with the negative outcomes of traumatic events [25]. Within psychology, there are several approaches to hope. According to Snyder [26], hope is a cognitive construct that reproduces a positive motivational state and the ability to strive toward own goals. To distinguish hope from similar constructs such as optimism and self-efficacy, Schneider [27] proposed that optimism derives from outcome expectancies, self-efficacy from agency expectancies and hope from both sets of positive expectations (wills and ways). Inside an alternative understanding with a more complex view of emotionality, [28] presented a holistic and integrative approach to hope, drawing on attachment, survival and mastery motives. Scioli and Biller [29] (p. 30), defined “hope as a future-directed, four-channel emotion network, constructed from biological, psychological and social resources”. Hope is both a state and a trait. As a trait, it is necessary to capture the wide spectrum of components that define it, such as biological systems, early environments, traits, centers of value and expressions. As a state, hope is an organizing and motivating collection of thoughts, feelings and actions.

Inside positive psychology, hope appears as a critical coping process (hoping) and a vital character strength (hopefulness). Furthermore, hope is the desired outcome, especially in the context of depression, disease, or other forms of dysregulation. Early research has demonstrated that hope is a protective factor that may positively impact a person’s psychosocial and spiritual development, including their perceived level of self-efficacy, resilience, effective coping and personal growth [30]. Although hopeful people believe that current situations can change for the better, those with strong self-efficacy believe they possess specific skills to make changes given a specific situation. In bad situations, individuals with a sense of high self-efficacy and higher hope are more inventive and reveal better determination in seeking their goals [31].

Joseph et collaborators [32] have found that hope is positively linked to people’s PTG. Conversely, a lack of hope may precipitate fear, depression, illness and impoverishment [33]. When hope is lost, there is no reason to work hard to leave hostile situations, because there is no way out.

Existing research has supported the negative link between hope and psychological health challenges. Hope has been associated with lower stress and depressive symptoms [34], a better quality of life [35], higher self-efficacy and resilience [36]. Hope is found to have a significant direct effect on satisfaction with life, positive affect and negative affect. Individuals with higher hope are more inventive and reveal better determination in seeking their goals [37].

### 1.3. Resilience

People throughout their lives are exposed to several stressful events that result in either positive or negative consequences. Resilience is considered as the capacity of individuals to cope with adverse life events successfully, resist illness and have the flexibility to adapt to new situations to maintain their psychological health; it represents a dynamic process that can increase the level of hope and positive attitude [38].

Previous studies have shown that resilience has a positive effect on a broad range of well-being outcomes, was positively related to satisfaction with life, affect balance and flourishing, and was negatively associated with negative affect [39,40]. Moreover, individuals who are more resilient demonstrate a better mental state and physical health, are able to adjust their level of control in consistent with the specific situation, perceive sufficient support from parents and can well buffer the adverse impact of traumatic events on the development of post-traumatic stress conditions [41].

## 2. Aim of the Study

Resilience, hope and self-efficacy may play a role in the relationship between symptoms of anxiety, stress and depression, and post-traumatic growth. To our knowledge, there is no study of the effect of these components on these relationships in adults concurrently. Therefore, the aim of this study was to test and verify the mediation of resilience, hope and self-efficacy and to analyze the moderating effect of gender and COVID-19 contagion on the association between symptoms of anxiety, stress and depression, and post-traumatic growth among adults during COVID-19.

**Figure 1:**
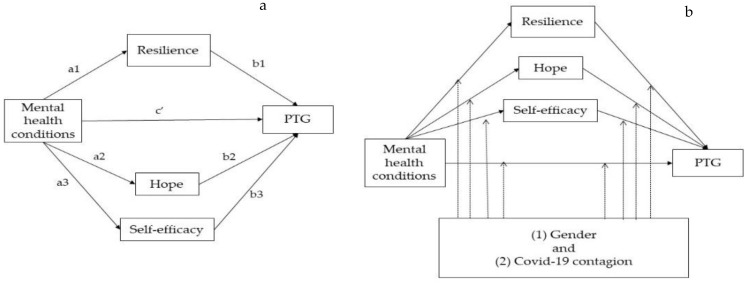
(**a**) Hypothesized mediation model explaining the potential mediating effect of resilience, hope and self-efficacy on the relationship between each of level of anxiety, depression and stress, and post-traumatic growth; (**b**) Model of the potential moderating effects on the paths. PTG: post-traumatic growth.

In our hypothesized model (Figure 1a), the total effects included a direct effect pathway (path c’) of each level of anxiety, depression and stress on the post-traumatic growth and a total indirect pathway (mediated: path a1b1+ path a2b2 + path a3b3) of each level of anxiety, depression and stress on the post-traumatic growth through resilience, hope and self-efficacy. Figure 1b shows the potential moderated association of gender and COVID-19 contagion in the multiple-mediation model.

Based on the theoretical support and explanations above, we proposed the following hypotheses:

**Hypothesis** **(H1).**  *Resilience, hope and self-efficacy would mediate the relationship between symptoms of anxiety, stress and depression, and post-traumatic growth.*

**Hypothesis** **(H2).**  *Gender and COVID-19 contagion would have a moderating effect on one or more paths among these variables.*

**Hypothesis** **(H3).**  *Post-traumatic growth would be negatively associated with anxiety, stress and depression.*

## 3. Materials and Methods

### 3.1. Eligibility Criteria

Eligibility criteria to take part in this study were the following: (1)–being 18 years old or older; (2) being available for all the time of data collection. Those who did not have internet access were excepted.

### 3.2. Procedure

An anonymous online questionnaire was prepared using an online survey platform (Google Forms, Google, Mountain View, CA, USA), and then distributed it through social media (i.e., Facebook, LinkedIn, Twitter, WhatsApp profiles) from 1 April to 21 July 2020. In Italy at that time there was a situation of lockdown starting 20 March 2020 until June with minor restrictions. The respondents came from multiple regions of Italy, spanning north and south. The time required to complete the survey took approximately 15 min. All respondents volunteered to participate in this study and signed a free informed consent after receiving a full description of the protocol of the study. Confidentiality and anonymity were ensured. The University Enna Kore Internal Review Board for psychological research approved the study [UKE-IRBPSY-07.21.01] and it was performed in line with the principles of the Declaration of Helsinki.

### 3.3. Participants

A total of 524 participated in the survey; *n* = 31 questionnaires were excluded because uncompleted, leaving 493 valid responses in the final dataset. The age of participants (*n* = 262 female and *n* = 231 male) ranges from 20 to 60 (*M_age_* = 33.40 years, SD = 13.41).

### 3.4. Measures

The survey predominantly consisted of the following two sections: the first one collected sociodemographic data (i.e., age, gender, level of education, marital/relationship status, occupation, financial burden and personal COVID-19 contagion). The second part of the survey included five questionnaires detailed as follows.

#### 3.4.1. Symptoms of Anxiety, Stress and Depression

The Italian version of the Depression Anxiety Stress Scales-21 [42] is a 21-item shortened version of the Depression Anxiety Stress Scale-42 [43], used to assess three negative emotional states (depression, anxiety and stress). The depression subscale includes dysphoria, hopelessness, anhedonia and lack of interest (Cronbach’s α = 0.81 for the scale in the current study); the anxiety subscale comprises situational anxiety, with somatic and subjective symptoms (Cronbach’s α = 0.75 for the scale in the current study); the stress subscale evaluates irritability, persistent arousal, psychological tension, hyperactivity, agitation (Cronbach’s α = 0.83 for the scale in the current study). A high score received from the scale is an indicator of disease. Responses were recorded by rating on a 4-point Likert scale, ranging from 1 (did not apply to me at all) to 3 (applied to me very much, or most of the time). Total scores for depression, anxiety and stress were calculated by aggregating their corresponding points. Each total was then doubled and then classified “normal”, “mild”, “moderate”, “severe” or “extremely severe”. The Italian DASS-21 demonstrated good criterion-oriented validity. Cronbach’s α = 0.95 for the scale in the current study.

#### 3.4.2. Posttraumatic Growth

The Italian adaptation [44] of the Posttraumatic Growth Inventory (PTGI) [45] included 21-items to assess positive outcomes reported by people who have experienced adverse and traumatic events through the following domains: spiritual change, appreciation of life, relating to others, new possibilities, personal strength. Response options range on a 6-point Likert scale from 0 (“*I did not experience this as a result of my crisis*”) to 5 (“*I experienced this change to a very great degree as a result of my crisis*”). Although the PTGI can be broken down into five subscales, the total score was used in the current study. The Italian version confirmed good criterion-oriented validity. Cronbach’s α detected in this sample was 0.96.

#### 3.4.3. Resilience

Resilience was assessed with the Italian version Resilience Scale [46], a 10-item questionnaire measuring resilience as the capacity to withstand the effects of life stressors, thrive from challenges and promote adaptation (e.g., *“I usually manage one way or another”; “I am determined”; “My life has meaning”; “When I am in a difficult situation, I can usually find a solution”*). Response options range from 1 (“strongly disagree”) to 7 (“strongly agree”), with higher scores reflecting higher levels of resilience. The Italian adaptation confirmed good psychometric properties and provided support for the construct validity of the scale. In the current sample, Cronbach’s α was 0.95.

#### 3.4.4. Self-Efficacy

The Italian version of the General Self Efficacy Scale [47,48] is a 10-items scale designed to assess optimistic self-beliefs to cope with demands in life and reach goals (e.g., *“Even if someone opposes, I can find the means and strength to achieve what I desire”; “It is easy for me to fixate on my goals and achieve them”; “I am confident that I can cope adequately with unexpected events”; “I can solve most problems if I invest adequate effort”*). Response options range on a 5-point Likert scale from 5 (“strongly agree”) to 1 (“strongly disagree”). Higher scores indicate a stronger sense of self-efficacy. The Italian version provided evidence for reliability and validity with minor modifications to the original version. In the present sample, Cronbach’s α was 0.93. I can succeed in solving difficult problems if I try hard.

#### 3.4.5. Hope

The Italian version of the Comprehensive State Hope Scale [28,49] is a 40-items questionnaire designed to measure state hope, including four larger sub-scales of *Spirituality* (spiritual inspiration and spiritual assurance); *Social support* (empowerment beliefs and interpersonal bonding); *Trust* (trust, openness and liberation beliefs); *Mastery* (goal progress and self-regulation). Response options are rated on 5-point scales with endpoints from 0 (“none”) to 4 (“extremely strong”). The total score (ranged from 17 to 141, skewness = 0.37, kurtosis = 0.12 and the Shapiro–Wilk statistic for normality = 0.99) was used in the current study. Cronbach’s α levels of reliability detected in this sample were adequate (α = 0.92).

### 3.5. Statistical Analysis and Sample Size

Data were expressed as means (*M*) ± standard deviations (*SD*) and the range. All statistical tests were two-tailed, and significance was determined at the 0.05 level. A *t*-test was used to detect the mean differences among sample. Additionally, Pearson’s correlation was used to determine the relationships between the selected variables. The scores of kurtosis and skewness were calculated to investigate normal univariate distribution. An a priori power analysis was run with G*Power [50]. This analysis found a multiple regression analysis with 23 predictors, an alpha level of 0.05 (two-tailed), a power of 0.80 to detect a small to medium effect size of = 0.06, required total sample size of *n* = 387. Therefore, a total of *n* = 493 participants were recruited to take part in the research.

A mediation analysis was performed to study whether the relationship between symptoms of anxiety, depression and stress (independent variables) and the post-traumatic growth (dependent variable) was mediated by resilience, hope and self-efficacy. To avoid biased parameter estimates, a multiple mediation model, which involves “simultaneous mediation by multiple variables” with Hayes’ (Hayes PROCESS version 4.0, Model 4–Ohio, Missouri, USA) computational tool [51] for SPSS was used in the current study.

Hence, we decided to test the moderating effect of gender and COVID-19 contagion among simple paths in the multiple-mediation model (symptoms of anxiety, depression and stress were displayed as predictors, post-traumatic growth as dependent variables, resilience, hope and self-efficacy as mediator, and COVID-19 contagion, and gender as covariates). A moderator analysis is used to specify whether both the direct and the indirect effects of the independent variable on the dependent variable can depend on the moderator value. Therefore, a moderated mediation analysis was conducted using Hayes’ (Hayes PROCESS version 4.0, Model 10–Ohio, Missouri, USA) computational tool for SPSS [51]. This tool enables the estimation of path coefficients, standard errors and different indexes of effect size, as well as the significance of the indirect effects obtained through the bootstrapping procedure with 5000 repetitions. The 95% confidence intervals (CI) for the coefficients calculated by bootstrapping methods were considered statistically significant if the confidence intervals did not include zero [52]. Statistical analyses were processed using SPSS version 25.0 (IBM, Armonk, NY, USA).

## 4. Results

### 4.1. Participants’ Characteristics

The sociodemographics characteristics of the participants are presented as counts and percentages (Table 1).

### 4.2. T-Test, Descriptives and Correlations

Table 2 shows the comparison between males and females, and COVID-19 contagion and no-COVID-19 contagion conducted through the Independent-samples *t*-test (*p* < 0.05).

As seen above, symptoms of anxiety, depression and stress, and self-efficacy were significantly higher in females than in males. Moreover, males and participants with COVID-19 contagion had significantly higher levels of resilience and post-traumatic growth. Symptoms of anxiety, depression and stress were significantly higher in participants with no-COVID-19 contagion than in participants with COVID-19 contagion.

The relationships among the variables of the study were analyzed using Pearson’s correlation coefficients. The means, standard deviations and correlations are presented in Table 3.

As can be observed, scores for resilience, hope and post-traumatic growth variables were significantly and negatively associated with scores for anxiety, depression and stress. On the other hand, scores for anxiety, stress and depression variables were significantly and positively related. There was also a positive relationship between self-efficacy and resilience. Hope was positively correlated with resilience and self-efficacy. Post-traumatic growth was positively correlated with resilience, self-efficacy and hope. Further investigation revealed the acceptable range of skewness and kurtosis values (from –1 to 1), representing a relatively normal distribution of each variable.

### 4.3. Mediation Model Analysis

**Figure 2:**
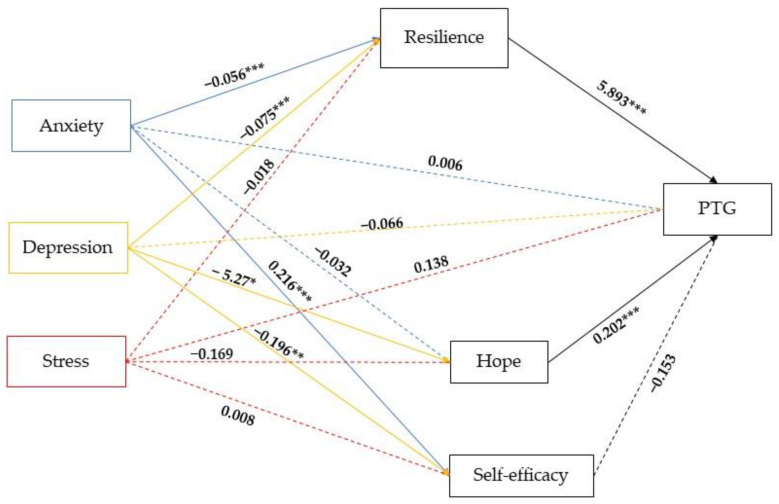
Parallel-multiple mediation of resilience, hope and self-efficacy between symptoms of anxiety, depression and stress, and post-traumatic growth (PTG). Note: * *p* < 0.05; ** *p* < 0.01; *** *p* < 0.001. Dashed lines indicate non-significant effects.

The results for the parallel mediating roles of three mediators (resilience, hope and self-efficacy) in the relationship between each level of anxiety, depression and stress, and post-traumatic growth are presented in Figure 2.

As presented in Figure 2, the total effect including a direct effect path-way (path c’) of anxiety (β = 0.006, SE = 0.199, *t*(486) = 0.031, *p* = 0.97), depression (β = −0.066, SE = 0.204, *t*(486) = −0.324, *p* = 0.75) and stress (β = 0.138, SE = 0.214, *t*(486) = 0.642, *p* = 0.52) on the post-traumatic growth was not statistically significant. The results also showed that not all of the simple path coefficients (mediated: path a1b1+ path a2b2 + path a3b3) were statistically significant with *p* < 0.05. The direct effects of anxiety on resilience (β = −0.056, SE = 0.013, *t*(489) = −4.207, *p* < 0.001) and self-efficacy (β = 0.216, SE = 0.08, *t*(489)= 2.694, *p* < 0.01), were statistically significant, as well as the direct effects of depression on resilience (β = −0.075, SE = 0.014, *t*(489) = −5.480, *p* < 0.001), hope (β = −0.527, SE = 0.24, *t*(489) = −2.163, *p* < 0.05) and self-efficacy (β = −0.196, SE = 0.08, *t*(489)= −2.364, *p* < 0.01). Furthermore, the direct effects of resilience (β = 5.893, SE = 0.70, *t*(486)= 8.353, *p* < 0.001) and hope (β = 0.202, SE = 0.04, *t*(486)= 4.887, *p* < 0.001) were statistically significant on post-traumatic growth.

The indirect effects of three mediators tested simultaneously are presented in Table 4, which contains the standardized β, indicating the intensity of the effect and the 95% CIs, indicating the significance of the effect with a 5% probability of error (CIs that do not contain 0 are significant).

Table 4 showed that the total indirect effects of anxiety on PTG trough resilience (β = −0.328, SE = 0.082, *p* < 0.001) and depression on PTG trough resilience (β = −0.443, SE = 0.100, *p* < 0.001) and hope (β = −0.106, SE = 0.060, *p* < 0.001), were statistically significant.

### 4.4. Moderated Mediation Analysis

Hence, we tested the moderating effect of gender and COVID-19 contagion among simple paths in the multiple-mediation model (symptoms of anxiety, depression and stress were displayed as predictors, post-traumatic growth as dependent variables, resilience, hope and self-efficacy as a mediator, and COVID-19 contagion and gender as moderators). Specifically, we run three moderated mediation models, considering the three independent variables. In Model 1, the independent variable was anxiety, while in Model 2, the independent variable was depression, and in Model 3, it was stress. The three moderated mediation analyses revealed that gender and COVID-19 contagion may impact symptoms of anxiety, depression and stress, and post-traumatic growth.

In Model 1, only the interaction of anxiety and COVID-19 contagion was statistically significant (β = 0.077 (0.022), *p* < 0.001, 95% CI [0.034, 0.120]), indicating that the relationship between anxiety and post-traumatic growth through resilience was moderated by COVID-19 contagion. Specifically, resilience was negatively associated to anxiety among women with no-COVID-19 contagion (β = −0.067 (−0.017); *p* < 0.001; 95% CI [−0.100, −0.034]—path a1). No significant relation was found in path a2. In path a3, the interactive effect of anxiety and gender (β = 0.342 (0.146), *p* < 0.05, 95% CI [0.056, 0.629]) and anxiety and COVID-19 contagion (β = 0.414 (0.133), *p* < 0.01, 95% CI [−0.675, −0.154]) on PTG via self-efficacy were statistically significant. Self-efficacy was negatively associated to anxiety among men with COVID-19 contagion (β = −0.430 (0.153); *p* < 0.01; 95% CI [−0.731, −0.129]); on the contrary, self-efficacy was positively associated to anxiety among women with no-COVID-19 contagion (β = 0.327 (0.102); *p* < 0.01; 95% CI [0.126, 0.527]).

A significant moderation effect emerged in the direct path from the anxiety to the post-traumatic growth. Specifically, anxiety was positively associated to PTG only among men with COVID-19 contagion (β = 1.386 (0.376), *p* < 0.001, 95% CI [0.647, 2.125]). The conditional indirect effect of anxiety on PTG through resilience (a1b1) was statistically significant (−0.295; 95% CI [−0.484, −0.144]) in the female group with no-COVID-19 contagion. The test of the index of moderated mediation indicated that only the moderated effect of COVID-19 contagion was significant (β = 0.340 (0.120); 95% CI [from 0.128 to 0.587]).

Additionally, in Model 2, only the interaction of depression and COVID-19 contagion was statistically significant (β = 0.039 (0.020), *p* < 0.05, 95% CI [0.000, 0.077]) indicating that the relationship between depression and post-traumatic growth through resilience was moderated by COVID-19 contagion. Specifically, resilience was negatively associated to depression among men with no-COVID-19 contagion (β = −0.064 (0.018); *p* < 0.001; 95% CI [−0.101, −0.028]—path a1) and women with no-COVID-19 contagion (β = −0.092 (0.017); *p* < 0.001; 95% CI [−0.126, −0.058]—path a1). In path a2, only the interactive effect of depression and gender (β = 0.982 (0.354), *p* < 0.01, 95% CI [0.286, 1.677]) on PTG via hope was statistically significant. Hope was negatively associated to depression among men with no-COVID-19 contagion (β = −0.970 (0.331); *p* < 0.01; 95% CI [−1.621, −0.320]) and among men with COVID-19 contagion (β = −1.473 (0.384); *p* < 0.001; 95% CI [−2.228, −0.719]). In path a3, the interactive effect of depression and gender (β = 0.426 (0.118), *p* < 0.001, 95% CI [0.195, 0.657]) and depression and COVID-19 contagion (β = −0.380 (0.116), *p* < 0.001, 95% CI [−0.608, −0.152]) on PTG via self-efficacy were statistically significant. Specifically, self-efficacy was negatively associated to depression among men (β = −0.726 (0.127); *p* < 0.001; 95% CI [−0.976, −0.475]) and women (β = −0.299 (0.126); *p* < 0.01; 95% CI [−0.546, −0.052]) with COVID-19 contagion. A significant moderation effect emerged in the direct path from the depression to the post-traumatic growth. Specifically, depression was positively associated to PTG only among men with COVID-19 contagion (β = 0.671 (0.324), *p* < 0.05, 95% CI [0.034, 1.308]). The conditional indirect effect of depression on PTG through resilience (a1b1) was statistically significant among men (−0.296; 95% CI [−0.522, −0.113]) and women (−0.424; 95% CI [−0.650, −0.237]) with no-COVID-19 contagion. The conditional indirect effect of depression on PTG through hope (a2b2) was statistically significant among men with no-COVID-19 contagion (−0.220; 95% CI [−0.416, −0.052]) and among men with COVID-19 contagion (−0.333; 95% CI [−0.587, −0.130]). The test of the index of moderated mediation indicated that only the moderated effect of gender was significant (β = 0.222 (0.095); 95% CI [0.048, 0.429]).

Finally, in Model 3, only the interaction of stress and COVID-19 contagion was statistically significant (β = 0.066 (0.020), *p* < 0.001, 95% CI [0.025, 0.106]), indicating that the relationship between stress and post-traumatic growth through resilience was moderated by COVID-19 contagion. Specifically, resilience was negatively associated to stress among women with no-COVID-19 contagion (β = −0.058 (0.022); *p* < 0.01; 95% CI [−0.102, −0.014]—path a1). No significant relation was found in path a2. In path a3, the interactive effect of stress and gender (β = 0.554 (0.134), *p* < 0.001, 95% CI [0.291, 0.817]) and stress and COVID-19 contagion (β = −0.369 (0.122), *p* < 0.001, 95% CI [−0.609, −0.128]) on PTG via self-efficacy were statistically significant. Specifically, self-efficacy was negatively associated to stress among men with COVID-19 contagion (β = −0.488 (0.130); *p* < 0.001; 95% CI [−0.743, −0.233]); on the contrary, self-efficacy was positively associated to stress among women with no-COVID-19 contagion (β = 0.435 (0.133); *p* < 0.01; 95% CI [0.173, 0.697]).

A significant moderation effect emerged in the direct path from the stress to the post-traumatic growth. Specifically, stress was positively associated to PTG only among men with COVID-19 contagion (β = 0.866 (0.327), *p* < 0.02, 95% CI [0.224, 1.509]). The conditional indirect effect of stress on PTG through resilience (a1b1) was statistically significant among women with no-COVID-19 contagion (−0.269; 95% CI [−0.524, −0.044]). The test of the index of moderated mediation indicated that only the moderated effect of COVID-19 contagion was significant (β = 0.303 (0.123); 95% CI [0.090, 0.568]).

## 5. Discussion

Our study aimed to test and verify the mediation of resilience, hope and self-efficacy and to analyze the moderating effect of gender and COVID-19 contagion on the association between symptoms of anxiety, depression and stress, and post-traumatic growth among adults during COVID-19. Firstly, our data showed that women reported higher scores on anxiety, stress and depression. These results support the existing literature on the role of gender as an important biological determinant of vulnerability to psychosocial stressors, concluding that women are more vulnerable to distress [53,54,55]. During the pandemic situation and the relative isolation characterized by a high degree of confusion, women are found to be more sad, anxious and stressed than men [56,57]. Moreover, women had greater chances of developing stress and feelings of emptiness impacting mental well-being because took care not only of their work responsibilities but also took care of the children and home, due to school closures during the pandemic [58,59,60]. Surprisingly, women also experienced higher levels of self-efficacy than men. This data differed from that of other studies that reported greater self-efficacy associated with lower stress and anxiety and a better quality of life [61,62]. Some studies instead reported a nonlinear and not exclusively positive relationship between increased self-efficacy levels and stress reduction [63,64]. Our results may be evaluated by considering the Yerkes-Dodson inverted-U model, suggesting that stress is beneficial but only up to a point at which it becomes damaging [65]. In fact, acute and chronic stressors can evoke different responses, also reflecting a more adaptive system or an evident hostile state. This position indicates that higher perceived self-efficacy does not always induce lower neuroendocrine reactivity and psychological adjustment. As the effects of self-efficacy are still dependent on a number of covariates, greater self-efficacy can also be linked to higher physiological reactivity [66]. In the current study, higher self-efficacy suggests that women might believe to have adequate abilities to cope with the negative impact of confinement in the context of the COVID-19 pandemic, maintaining relatively stable emotions even under pressure.

Moreover, participants with no-COVID-19 contagion reported higher scores on stress, anxiety and depression. Potentially, the imposed home-confinement strategy, the fear of COVID-19 and related uncertain consequences of contagion might have heightened the personal insecurity. This consideration is in line with other studies that have reported people’s fears about their own health or fears of infecting other people [67,68].

The parallel mediating roles of three mediators (resilience, hope and self-efficacy) in the relationship between each level of anxiety, depression and stress, and post-traumatic growth partially confirmed H2. Anxiety was negatively associated with PTG indirectly via resilience. Moreover, depression was negatively associated with PTG through resilience and hope. The inverse association between resilience and PTG is added to previous evidence indicating a curvilinear relationship in which growth is highest at moderate levels of anxiety, depression and stress [69]. Hope and resilience are significant components of mental well-being, orienting individuals to have beliefs that they can cope with stressful events. During the current situation, in which the predominant feeling was fear, the ability to plan future goals and to use ways of reaching one’s goals was very important in reducing anxiety and depression. Furthermore, in stressful times, high hope may promote many resources that will stimulate the PTG [70].

These findings highlighted the significant role of personal resources (e.g., hope and resilience) and revealed ways to increase psychological growth even under pressured circumstances [71,72].

In the last analysis, we tested the moderating effect of gender and COVID-19 contagion among simple paths in the multiple-mediation model (symptoms of anxiety, depression and stress were displayed as predictors, post-traumatic growth as dependent variables, resilience, hope and self-efficacy as mediators). Partially in line with H3, results revealed that gender and COVID-19 contagion may impact symptoms of anxiety, depression and stress, and post-traumatic growth. In each of the three models, a moderating effect of COVID-19 contagion emerged on indirect associations between anxiety, depression and stress, and post-traumatic growth, via resilience. Precisely, in the female group with no-COVID-19 contagion, resilience decreased with increasing anxiety, depression and stress; in the male group with no-COVID-19 contagion resilience decreased with increasing depression. In sum, while the evidence suggests that women in normal situations experience more stress, the non-exposure to the virus leads individuals to be more insecure and vulnerable to the psychosocial effects of the pandemic [73]. Moreover, in all three models, moderating effects of gender and COVID-19 contagion emerged on the indirect association between anxiety, depression and stress, and post-traumatic growth, via self-efficacy. Specifically, among men with COVID-19 contagion self-efficacy decreased with increasing anxiety and stress. Unexpectedly, in women with no-COVID-19 contagion self-efficacy increased with increasing anxiety and stress. Lastly, among men and women with COVID-19 contagion self-efficacy decreased with increasing depression. We saw that self-efficacy can lead to decreases and increases in parameters of the physiological stress response. As the effects of self-efficacy are partly dependent on a number of covariates, the question as to whether the efficacy-stress-reaction-relationship should be positive or negative is related to individual differences. We can also conclude that an improved perception of one’s efficacy does not widely reduce the stress response [74].

A moderating effect of gender emerged on the indirect association between depression and post-traumatic growth through hope. Specifically, among men with COVID-19 contagion and no-COVID-19 contagion, hope decreased with increasing depression. This result converges with some cross-sectional studies that found low levels of hope associated with elevated anxiety, depression and post-traumatic stress disorder [75,76].

In each of the three models, a significant moderation effect emerged in the direct path from anxiety, depression and stress to post-traumatic growth. Specifically, anxiety, depression and stress were positively associated with PTG only among men with COVID-19 contagion. These findings are in line with [77], showing that higher levels of positive growth were reported by individuals also experiencing a higher level of anxiety (*r* = 0.53). Similarly, Silva et colleagues [78] reported a significant positive association (*r* = 0.34) between symptoms of depression and PTG six months following a traumatic event. Authors suggest that higher levels of psychological distress may promote re-evaluation of one’s priorities and values post-trauma.

The correlational data, revealing that scores for resilience, hope and post-traumatic growth variables were significantly and negatively associated with scores for anxiety, depression and stress, confirmed the initial hypotheses (H1). Post-traumatic growth was positively correlated with resilience, self-efficacy and hope. These findings are consistent with those of previous studies, confirming that resilient, self-efficacious and hopeful individuals are more likely to overcome adversity and can deal with potential stressors effectively. Therefore, it can be said that these people are optimists, have high control over their lives and achieve PTG more quickly [79,80,81].

## 6. Limitations of the Study

In interpreting the findings, several limitations of this study should be considered. First, the study design is cross-sectional and, therefore, no conclusions about the direction of relationships are possible. Future longitudinal studies would be helpful to further understand patterns of hope, self-efficacy, resilience and PTG. Second, although the participants came from all geographical areas of Italy, the sample size is quite small. Future research with a large and representative sample would enrich and strengthen the evidence that is documented in the current study. Third, people reported their participation through self-reported measures, which could be contaminated with optimistic bias. Moreover, on the issue of generalization, this research study was conducted in Italy. It would be interesting to replicate the study in other countries. Future research on developing effective public health interventions to promote participation in health-protective behaviors could help to intensify infection control and increase pandemic alertness in the future.

## 7. Conclusions

Despite some methodological limitations, the consistency of the statistical results supports the fact that the present study contributed to increasing knowledge of the psychological impact of the COVID-19 pandemic in Italy. Study findings provide novel evidence of the association between symptoms of anxiety, depression and stress, and post-traumatic growth in a distress-related situation. Statistical analyses indicated that anxiety and depression may indirectly impact PTG via the mediating role of resilience and hope. At the same time, it was further tested that the relationship between symptoms of anxiety, depression and stress is moderated by gender and COVID-19 contagion. The study also provided evidence that females were more vulnerable to psychological distress. Potential applications of these findings may be to integrate the development of resilience and hope as intervention targets to encourage people to find ways out of their adverse experiences.

From our point of view, it requires the development of specific actions for the management of fear of COVID-19, supporting both people that have been infected or continue to have social restrictions (i.e., vulnerable people). The COVID-19 outbreak can be an opportunity for each affected country to better set up its preparedness for future health emergencies while also foreseeing appropriate initiatives to prevent psychological distress in the population.

## Figures and Tables

**Table 1:** Participants sociodemographics characteristics, (*n* = 493).

Characteristic	Frequency (*n*)	Percentage (*%*)
**Gender**		
Male	231	(46.9)
Female	262	(53.1)
Education		
High School	293	(59.4)
Graduate degree	200	(40.6)
Relationship status		
Unmarried	363	(73.6)
Married	103	(20.9)
Separated/Divorced	27	(5.5)
Occupation		
Employed	198	(40.2)
Student	295	(59.8)
Economic situation after COVID		
Worsened	107	(21.7)
Unchanged	352	(71.4)
Improved	34	(6.9)
COVID-19 contagion		
Yes	189	(38.3)
No	304	(61.7)

**Table 2:** Differences among sample from male vs. female, and from COVID-19 contagion vs.no-COVID-19 contagion on dimensions of the study.

		Gender					COVID-19 Contagion				
Dimension		Male	Female	*t*	*p*	*Cohen’s d*	Yes	No	*t*	*p*	*Cohen’s d*
Anxiety	*M*	7.35	14.42	−14.13	<0.001	−1.275	7.95	13.07	−9.09	<0.001	−0.842
	*SD*	3.99	6.60				4.92	6.69			
Depression	*M*	7.81	14.63	−12.20	<0.001	−1.101	8.31	13.38	−8.25	<0.001	−0.764
	*SD*	5.55	6.71				5.60	7.19			
Stress	*M*	10.05	16.92	−13.74	<0.001	−1.24	11.31	15.19	−6.71	<0.001	−0.621
	*SD*	5.41	5.65				5.55	6.63			
Resilience	*M*	5.08	3.84	9.70	<0.001	0.875	5.07	4.02	7.80	<0.001	0.722
	*SD*	1.43	1.40				1.32	1.54			
Hope	*M*	83.71	82.50	0.60	0.55	0.054	84.08	82.43	0.79	0.426	0.074
	*SD*	23.40	21.40				22.76	22.09			
Self-efficacy	*M*	35.03	36.98	−2.90	<0.001	−0.262	35.20	36.60	−2.03	0.043	−0.188
	*SD*	8.45	6.44				7.94	7.18			
Post-traumatic growth	*M*	50.86	39.79	6.12	<0.001	0.552	49.99	41.86	4.30	<0.001	0.398
	*SD*	22.77	17.27				23.71	18.05			

**Table 3:** Descriptive statistics and Pearson correlation coefficients related to variables.

Variable	1	2	3	4	5	6	7
1. Anxiety	1						
2. Depression	0.728 **	1					
3. Stress	0.723 **	0.785 **	1				
4. Resilience	−0.541 **	−0.575 **	−0.516 **	1			
5. Self-efficacy	0.059	−0.042	−0.001	0.281 **	1		
6. Hope	−0.166 **	−0.212 **	−0.187 **	0.329 **	0.430 **	1	
7. Post-traumatic growth	−0.259 **	−0.283 **	−0.239 **	0.483 **	0.162 **	0.334 **	1
Mean	11.11	11.43	13.70	4.42	36.06	83.07	44.98
Standard Deviation	6.559	7.062	6.510	1.542	7.502	22.347	20.765
Skewness	0.336	0.250	−0.178	−0.104	−0.540	0.098	0.821
Kurtosis	−1.160	−1.263	−1.324	−1.078	0.309	−0.170	−0.151

Note. ** *p* < 0.001.

**Table 4:** Summary of indirect effects and confidence intervals of three mediators (N = 493).

Mediators	Indirect Effects	SE	Z	*p*	Boot LLCI	Boot ULCI
Anxiety→ Resilience → PTG	−0.328	0.082	−3.83	<0.001	−0.496	−0.171
Anxiety → Self-efficacy → PTG	−0.033	0.032	−1.11	0.26	−0.102	0.025
Anxiety→ Hope → PTG	−0.007	0.049	−0.13	0.89	−0.108	0.090
Depression → Resilience → PTG	−0.443	0.100	−4.51	<0.001	−0.654	−0.259
Depression → Self-efficacy → PTG	0.030	0.031	1.08	0.27	−0.018	0.103
Depression → Hope → PTG	−0.106	0.060	−4.80	<0.001	−0.238	−0.004
Stress → Resilience → PTG	−0.103	0.087	−1.18	0.23	−0.278	0.065
Stress → Self-efficacy → PTG	−0.001	0.018	−0.08	0.92	−0.043	0.035
Stress → Hope → PTG	−0.034	0.050	−0.63	0.52	−0.136	0.064

## Data Availability

The data that support the findings of this study are available from the corresponding author (D.D.C.), upon reasonable request.
